# Keratosis Pharyngis

**Published:** 1951-01

**Authors:** E. Watson-Williams


### KERATOSIS PHARYNGIS

BY

E. WATSON-WILLIAMS, *M.C.*, M.D., CH.M.

---

Mrs. Q. M. M., 30, noticed " spots " on her throat and tongue four months ago. She was and is quite well: " the tongue felt uncomfortable " and she then saw the spots more or less as they are now—prominent, irregular, greyish masses on either tonsil, mainly round the crypta magna, on the posterior part of the tongue and on the back wall of the pharynx. Some are fully 1 cm. long, others much smaller; and all firmly adherent—i.e. they are not crypt debris, pus or " membrane ". Some discomfort—no pain.

This condition is not very rare: in some thirty years I have recorded fifty-seven cases, forty-four in females. The cause is obscure. The masses may be few and small, and if confined to the tonsils produce an appearance readily mistaken for follicular tonsillitis. (The absence of fever, pain, and fetor should prevent this mistake: the firm attachment of the masses and their persistence show their true nature—often the patient will say: " That's not tonsillitis, doctor, my throat's been like that for years ".) They are formed of thickened, keratinised, squamous epithelium, are sometimes lobulated or pedunculated, and when large are often stained brown or black by fungi. Some patients get repeated attacks, the masses appearing if they are over-tired or run down: a girl of eleven had had three such attacks beginning each autumn with urticaria and a few days' malaise, and lasting three to four months. In others there is little or no change in appearance over several years—and then, for no obvious reason, the masses may disappear quite suddenly. I have one family: father, son and daughter all being affected. There is no pain, though sometimes some discomfort : but often the condition is discovered accidentally during routine examination.

*Treatment*. If the masses are confined to the tonsils, and causing discomfort, removal of the tonsils may be desirable. Curetting or cauterizing often seems to stimulate recurrences. The patient should be reassured and given a short account of the natural history. A long course of arsenic is said to be helpful: but as so often the condition resolves spontaneously it is difficult to assess the value of medicines.

Patient, and specimens shown at the Clinical Meeting, 8th November, 1950.

